# Cytology and Histology of Thyroid Nodules: Exploring Novel Insights in the Molecular Era for Enhanced Patient Management

**DOI:** 10.3390/curroncol30080562

**Published:** 2023-08-21

**Authors:** Beatrix Cochand-Priollet, Zahra Maleki

**Affiliations:** 1 Department of Pathology, Cochin AP-HP-Paris Centre, University Paris-Cité, 75679 Paris, France; 2 Department of Pathology, Johns Hopkins Hospital, The Johns Hopkins Medical Institution, Baltimore, MD 21287, USA; zmaleki1@jhmi.edu

**Keywords:** thyroid nodule, WHO, *The Bethesda System for Reporting Thyroid Cytopathology*, terminology, thyroid cancer, biomarkers

## Abstract

Significant advancements have been made over the past decade in our understanding of thyroid cancers, encompassing histomorphology, cytology, and ancillary techniques, particularly molecular tests. As a result, it is now feasible to put forth a comprehensive histo/cytomolecular approach to treating these tumors, thereby offering patients treatments that are precisely tailored to their unique circumstances.

## 1. Introduction

Over the past decade, the landscape of thyroid neoplasm diagnosis has undergone a significant transformation. The catalyst for these changes emerged in 2016 with the introduction of a novel entity termed “NIFTP.” This groundbreaking development was not initially unveiled within a scientific journal, but rather within the pages of *The New York Times* on 14 April 2016. Accompanied by a striking histological image of a completely encapsulated microfollicular nodule, the article bore the title “It’s not a cancer: doctors reclassified a thyroid tumor”. The subsequent explanation clarified that it pertained to “a non-invasive thyroid follicular neoplasm with papillary-like nuclear atypia”, known as NIFTP. This particular type of tumor had formerly been regarded as a form of cancer, but was re-evaluated, downgraded, and reclassified by a consortium of medical experts. Unquestionably, this article acted as a catalyst, sparking a paradigm shift across various medical disciplines—endocrinology, surgery, radiology, oncology, and more—involved in the oversight and treatment of thyroid nodules. Equally affected were the patients, and prominently, the pathologists who stood on the frontlines of these changes. The principle scientific article on this subject was published in August 2016 in the journal *JAMA Oncology*, featuring Yuri E. Nikiforov as the first author [1].

**Figure 1:**
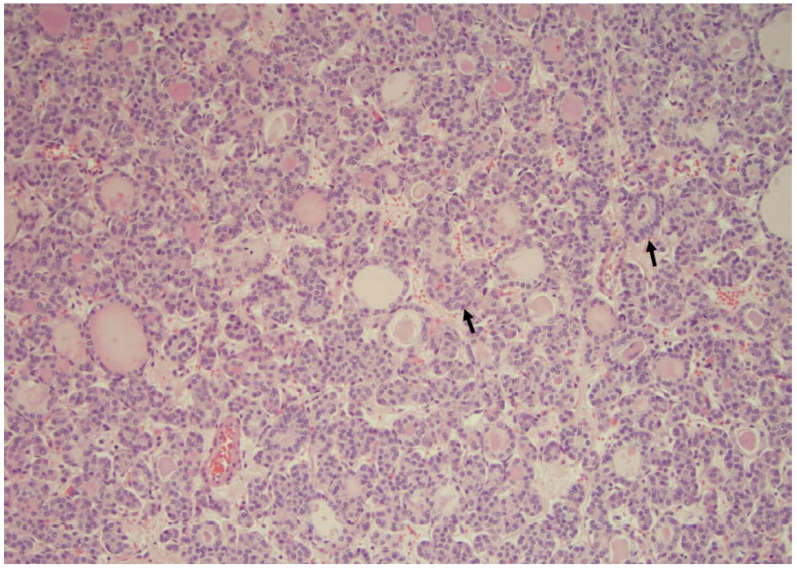
NIFT-P is characterized by a microfollicular architecture and some nuclear atypia suggestive of a papillary carcinoma (arrows) (×200, H&E stain).

The main histological criteria were outlined in the original research text, garnering significant attention. In the year 2017 alone, around 87 studies were subsequently published, which can be found on PubMed. The researchers of these studiesconcluded that thyroid tumors currently diagnosed as non-invasive EFVPTC (encapsulated follicular variant of papillary thyroid carcinoma) pose a very low risk of adverse outcomes and should be designated as NIFTP (Figure 1). They postulated that this reclassification would impact the global patient population, leading to a marked reduction in psychological and clinical implications linked to a cancer diagnosis. Therefore, it was explicitly indicated that NIFTP is not a novel entity but rather the outcome of a prognostic evaluation based on the insights of an extensive panel of thyroid tumor experts and a thorough, long-term assessment of its actual risk of malignancy (ROM). Indeed, addressing the concern of overdiagnosis and overtreatment of indolent cancers in various organs, the National Cancer Institute orchestrated a conference in 2012 to deliberate on this pivotal issue. Subsequent to the conference, an international group of expert pathologists and clinicians was assembled in order to analyze the thyroid tumors known to have a low ROM. The identification of NIFTP swiftly prompted its incorporation into the fourth edition of the WHO classification of thyroid glands [2] in 2017. In this classification, NIFTP found its place within a category termed “Encapsulated Follicular tumors”. This category encompassed not only NIFTP but also other tumors, such as follicular tumors of uncertain malignant potential (FTUMP) and well-differentiated follicular tumors of uncertain malignant potential (WDFTUMP).

Due to an enhanced understanding of the molecular makeup of thyroid tumors and a deeper understanding of the biological significance of their biomarkers, the fifth edition of the WHO classification was recently unveiled in 2022. Notably, the period spanning from 2017 to 2022 witnessed the positive influence of the European Thyroid Association’s guidelines for ultrasound-based malignancy risk stratification in adult thyroid nodules, known as “the EU-TIRADS” [3]. Within this timeframe, a re-evaluation of *The Bethesda System for Reporting Thyroid Cytopathology* (*BSRTC*) became imperative, leading to the impending release of its third edition in 2023 [4,5]. Consequently, a profound transformation in the management of patients with thyroid nodules is anticipated, relying on intricate algorithms that encompass clinical data, ultrasound characteristics, cytological classifications, and molecular markers. The objective of this review is to outline the most recent and noteworthy advancements in the realms of histology and cytology, and how these developments impact patient management.

## 2. WHO Classification of Thyroid Tumors, 5th edition, 2022

As previously discussed, the updated WHO classification [6] represents a fusion of molecular and histopathological aspects. The histoprognostic classification for established tumors has been revised based on an enhanced understanding of the molecular aberrations frequently associated with each tumor type. This integration of histological and molecular insights has precipitated a series of significant modifications, which are extensively elucidated and documented in Zubair Baloch’s comprehensive review titled “Overview of the 2022 WHO classification of thyroid neoplasms” [7].

(A) Benign follicular tumors

This category encompasses follicular adenoma, a newly recognized benign entity termed “follicular adenoma with papillary architecture,” and oncocytic adenoma. In instances of multiple benign nodules, the term “goiter” is no longer recommended. Instead, it has been replaced with the term “Thyroid Follicular Nodular Disease (TFND).” “Follicular adenoma with papillary architecture” characterizes an encapsulated neoplasm featuring centripetal papillary structures devoid of nuclear atypia. It is often associated with McCune–Albright syndrome and Carney complex, which exhibit distinct molecular alterations. The adoption of “TFND” aims to avoid the terminology of “hyperplastic” or “neoplastic,” recognizing that solely based on morphology, differentiation between clonal and metabolic disorders is challenging.

(B) Low-risk follicular thyroid neoplasms

These include non-invasive follicular thyroid neoplasms with papillary-like nuclear features (NIFTP), thyroid tumors of uncertain malignant potential (UMP), and hyalinizing trabecular tumors (HTTs). All these tumors are characterized by their indolent behavior and a lack of BRAF V600E mutations.

Regarding NIFTP, as described in the Introduction, tumors with a diameter ≤1 cm are now classified as NIFTP in the WHO 2022 classification (previously being designated as the PTC subtype in WHO 2017). UMP tumors are encapsulated follicular tumors with unclear capsular invasion. They are labeled “follicular tumor UMP” when lacking nuclear atypia, and “well-differentiated follicular tumor UMP” when exhibiting some PTC-like nuclear atypia. HTTs are characterized by a distinct morphology featuring a trabecular arrangement, hyaline material, and markedPTC-like atypia. Recent findings associate HTTs with specific GLIS gene rearrangements, without BRAF or RAS mutations.

(C) The differentiated follicular-cell-derived thyroid carcinoma

C-1—The most frequently occurring carcinomas are papillary thyroid carcinomas (PTCs) and follicular thyroid carcinomas (FTCs), which are classically characterized by their architecture and the presence or lack of nuclear atypia. These malignancies are also delineated by their molecular profiles. PTC, regardless of its architecture, papillary, or other morphological variants, is characterized by the BRAF V600E mutation, while FTC is associated with RAS mutations, underscoring the alignment between morphology and molecular signature.

Concerning the follicular variant of PTC, it is now confirmed that the encapsulated variant (EFVPTC) is a RAS-like neoplasm and the invasive variant (IFVPTC) is a BRAF-like tumor [8].

C-2—The term “Papillary microcarcinomas (micro-PTC)” has been eliminated and replaced with the term “papillary carcinomas”, which is to be employed irrespective of the tumor’s size, even if it measures less than 1 cm in diameter Previously, “micro-PTCs” were categorized separately due to their generally favorable prognosis. However, recent studies have revealed that these micro-PTCs possess a metastatic risk depending on their histological subtypes and their location within the thyroid gland [9]. As a result, it is now recommended that micro-PTCs no longer be considered a distinct subtype of PTC.

C-3—The PTC subtypes. 

**Figure 2:**
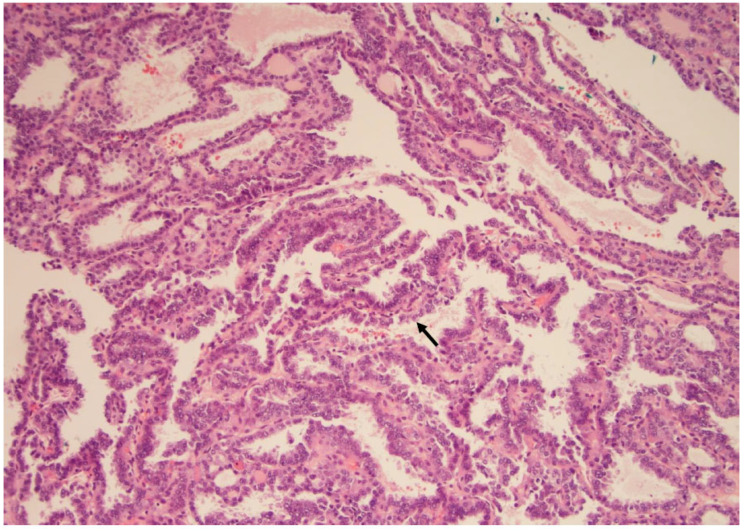
The classic type of papillary carcinoma is entirely composed of papillary structures, i.e., a central fibrous core lined by atypical cells (arrow), as seen here (×200, H&E stain).

**Figure 3:**
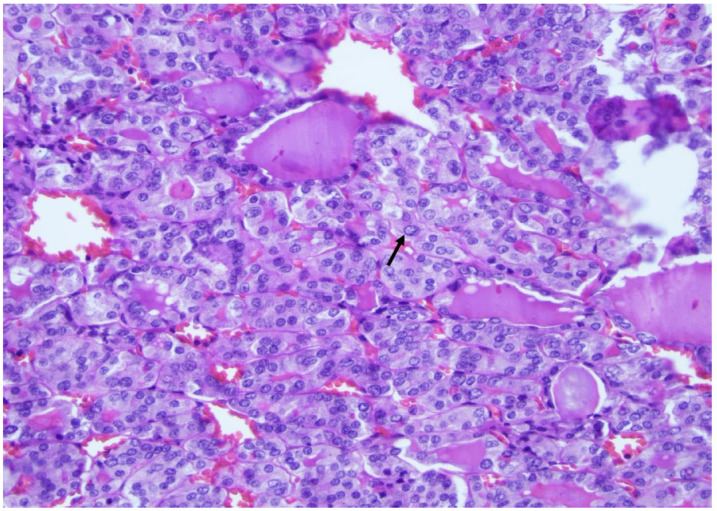
The papillary carcinoma follicular subtype is characterized by a microfollicular architecture with marked nuclear atypia suggestive of a papillary carcinoma like the intranuclear pseudoinclusion (arrow) (×200, H&E stain).

**Figure 4:**
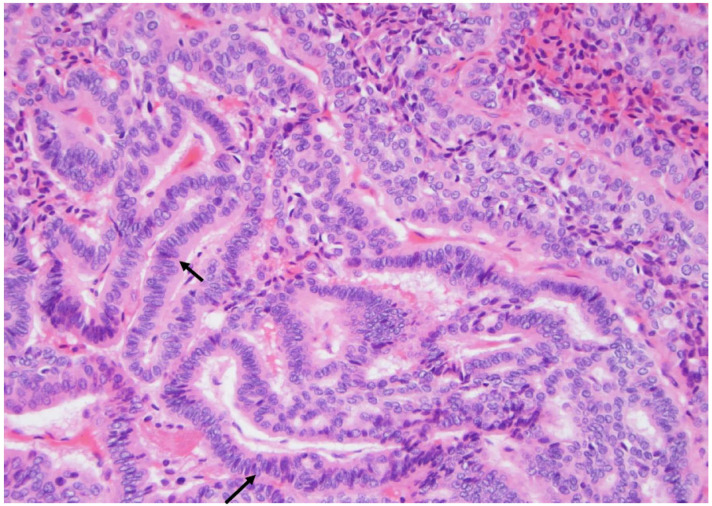
The papillary carcinoma tall cell subtype displays the same architecture as the papillary carcinoma type, but the cells are taller and their height is at least three times their width, and they exhibitabundant cytoplasm and frequent intranuclear pseudoinclusions (arrows) (×200, H&E stain).

**Figure 5:**
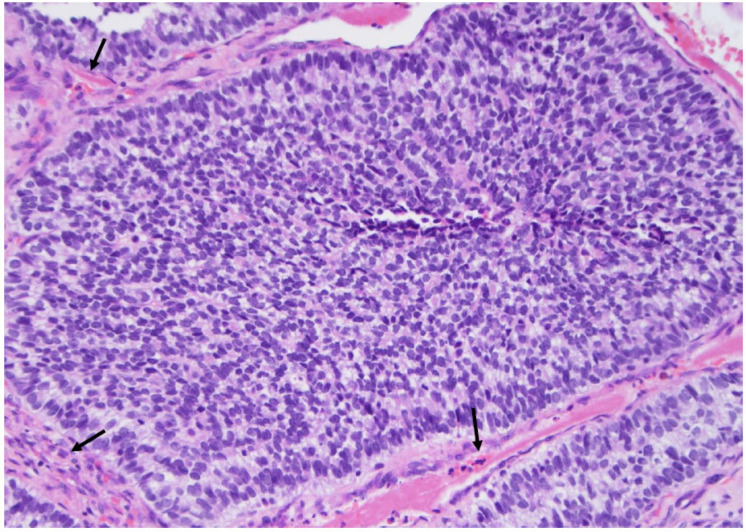
The papillary carcinoma trabecular subtype does not show any papillary or follicular architecture but a more solid/nested architecture and marked nuclear atypia. Note the malignant cells in large nests separated by a network of connective tissue and blood vessels (arrows) (×200, H&E stain).

In a concise overview, particular attention should be directed towards identifying certain PTC subtypes, including the tall cell, columnar cell, and hobnail variants, due to their more aggressive behavior when compared to classic PTC (Figure 2, Figure 3, Figure 4 and Figure 5). These subtypes are linked to mutations in the TERT promoter, TP53, and PIK3CA genes. Similarly, the diffuse sclerosing and solid/trabecular subtypes are also categorized as more aggressive tumors. It is imperative we recognize and distinguish these subtypes accordingly. Conversely, the prognosis is not adversely affected by oncocytic PTC, Warthin-like, and clear cell subtypes. 

The classification of cribriform-morular thyroid carcinoma has been revised. It is no longer regarded as a subtype of PTC; instead, it has been established that this tumor, often diagnosed in individuals with familial adenomatous polyposis (FAP), is not associated with BRAF V600E or RAS mutations, but rather APC mutations. Consequently, these cancers can now be identified through nuclear and cytoplasmic beta-catenin immunopositivity on immunohistochemistry. Moreover, considering its negative immunostaining for TTF1 and thyroglobulin markers, cribriform-morular thyroid carcinoma has been repositioned within the category of “thyroid tumor of uncertain histogenesis”.

C-4—The term “ Hürthle cell” has been discarded as it is now widely recognized that oncocytic cells are distinct from Hürthle cells, and the retention of this term was primarily due to historical tradition in the USA. It is now recommended that more accurate terminology be used, i.e., “oncocytes” and “oncocytic”. Moreover, the 2017 WHO classification paved the way for oncocytic tumors to be categorized as a separate entity. Oncocytic carcinoma refers to malignant tumors comprising over 75% oncocytic cells. It is important to differentiate oncocytic carcinoma from oncocytic PTCs, a task that can be challenging based solely on morphological characteristics. Consequently, molecular testing is of value in differentiating between the two, with the former subtype being characterized by changes in the mitochondrial genome or the GRIM 19 gene and copy number variations, and the latter by the BRAF V600E mutation.

**Figure 6:**
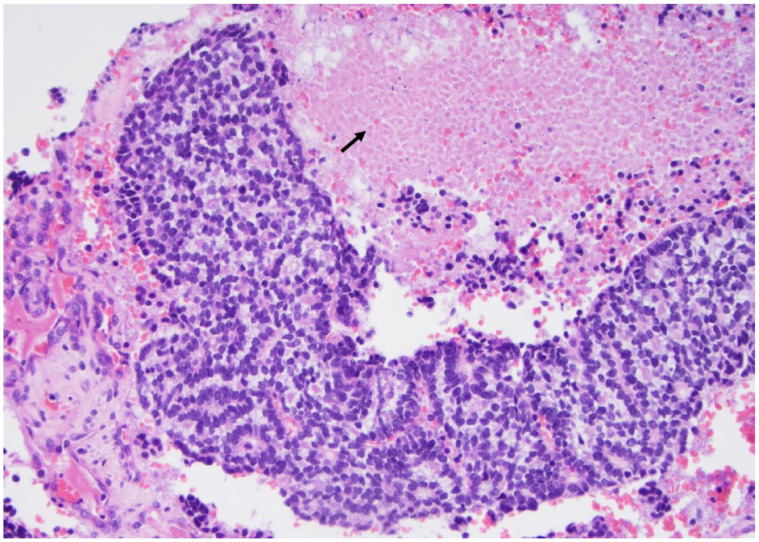
High-grade carcinoma; the same tumor with trabecular and solid patterns with an area of extensive necrosis (arrow) (×200, H&E stain).

C-5—A new group of high-grade thyroid carcinomas has been added in the 2022 WHO classification. This group includes poorly differentiated thyroid carcinomas (PDTCs) already known and defined by the Turin criteria including solid/trabecular growth, convoluted nuclei, tumor necrosis, and/or a mitotic count ≥ 3 mitoses/2 mm^2^ (Figure 6).

All the well-differentiated carcinomas listed above, i.e., PTC and its subtypes, FTC, and OTC, are now classified as high-grade differentiated thyroid carcinoma (HGDTC) in cases of increased mitotic activity (greater than 5 mitoses/2 mm^2^) and/or tumor necrosis.

For all these tumors, the overall 10-year survival is lower compared to that for the other differentiated carcinomas. Most of these tumors have driver BRAF V600 E or RAS mutations, but secondary mutations in the TERT promoter, TP 53, or PIK3CA can also be found.

(D) The anaplastic carcinoma

**Figure 7:**
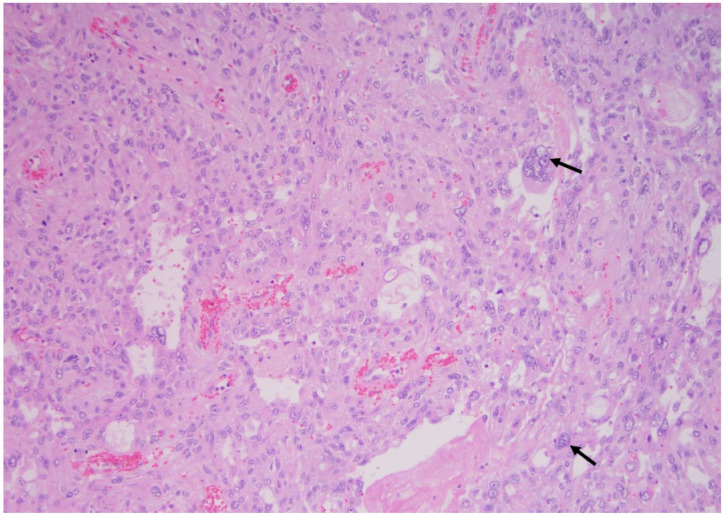
Anaplastic carcinoma with large, more or less cohesive cells without any specific architecture; note the large multinucleated epithelial cells (arrows) (×200, H&E stain).

**Figure 8:**
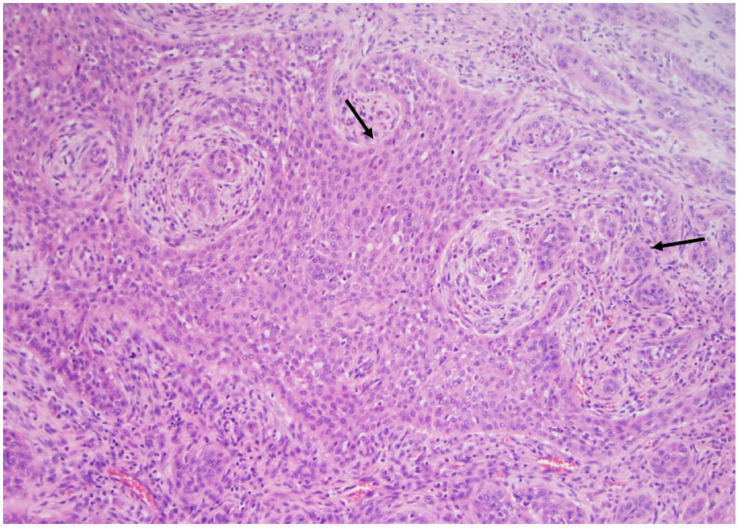
Squamous cell carcinoma is characterized by sheets and ribbons (arrows) of cohesive cells with variable degrees of differentiation, (×200, H&E stain).

The diagnostic morphological criteria for this carcinoma did not change from 2017 to 2022. It is a very rare yet highly aggressive cancer (Figure 7). Nevertheless, it is now recommended these carcinomas be promptly tested for the BRAF V600E mutation since the efficacy of the targeted therapies has been proven, even for anaplastic carcinoma. Moreover, the very rare squamous cell carcinoma (Figure 8), pure or associated with another type of carcinoma, should be regarded as the equivalent of an anaplastic component and managed accordingly.

(E) Medullary thyroid carcinomas or derived thyroid C-cell carcinoma (MTC)

**Figure 9:**
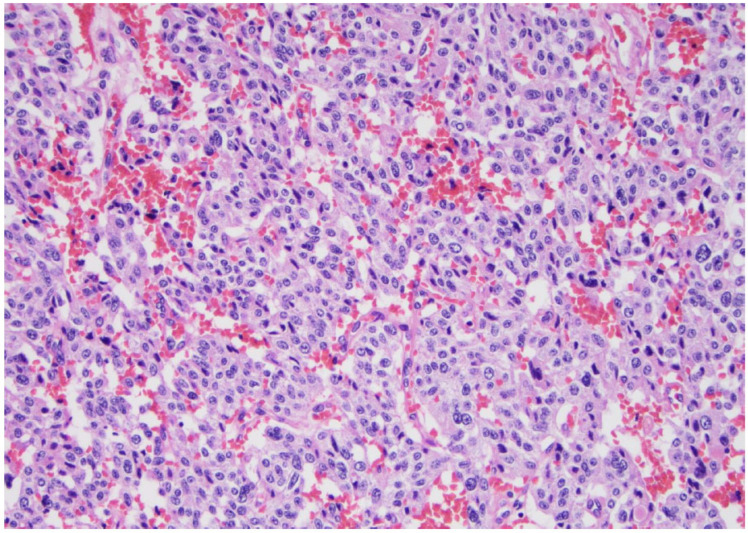
Medullary carcinoma shows lobular or nested groups of medium-sized cells with relatively round or oval uniform nuclei with scattered nuclear variations (×200, H&E stain).

**Figure 10:**
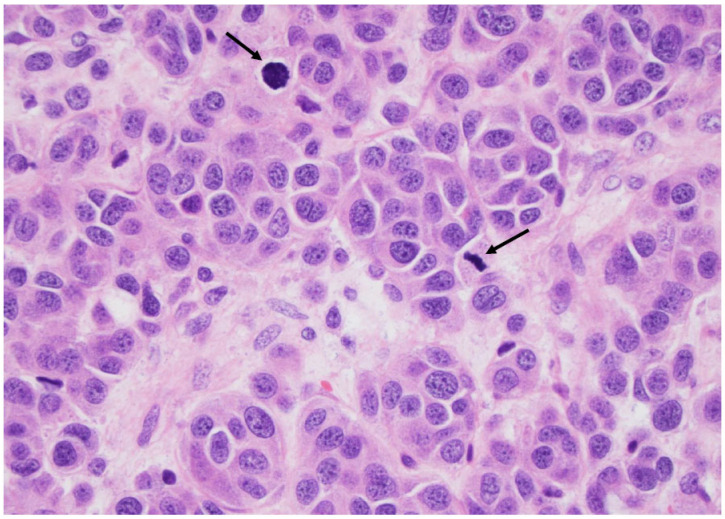
Medullary thyroid carcinoma at a higher magnification: note the mitosis (arrows), indicating a high-grade medullary carcinoma diagnosis (×200, H&E stain).

**Figure 11:**
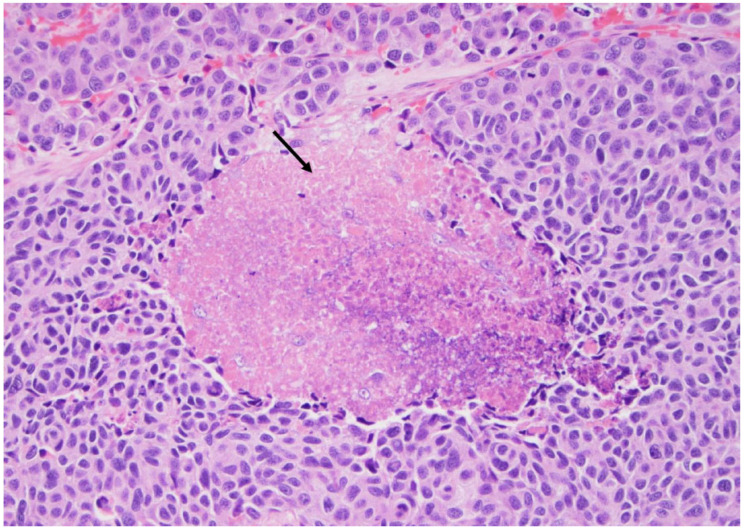
Medullary thyroid carcinoma: note the necrotic area (arrows), indicating a high-grade medullary carcinoma diagnosis (×200, H&E stain).

The most significant update for medullary thyroid carcinoma (Figure 9) is the introduction of a grading system called “the International medullary thyroid carcinoma grading scheme”. This is a two-tiered grading system; high-grade cancers are characterized by tumor necrosis; a mitotic count ≥ 5 mitoses per 2 mm^2^; and/or a Ki 67 proliferation index ≥ 5% (Figure 10 and Figure 11) [10].

(F) Rare tumors

Rare tumors are included and described in the WHO 2022 classification but are not discussed here due to their rarity.

## 3. The Bethesda System for Reporting Thyroid Cytology, 3rd Edition, 2023

In the third edition of *The Bethesda System for Reporting Thyroid Cytology* [4,5], whose editors are Syed Z Ali, from the Johns Hopkins University School of Medicine in Baltimore, USA, and Paul A VanderLaan from Beth Israel Deaconess Medical Center and Harvard Medical School, Boston, USA, the main objectives are as follows:

To standardize the terminology for the six already-known categories of *The BSRTC*;

To propose a more precise assessment of the risk of malignancy (ROM) for each category;

To be in agreement with the WHO 2022 classification;

To include all the data available for better management of each patient.

Therefore, the changes to the terminology, the ROM for each category, and the patient management along with those terms that were retained are presented in the following table (Table 1).

**Figure 12:**
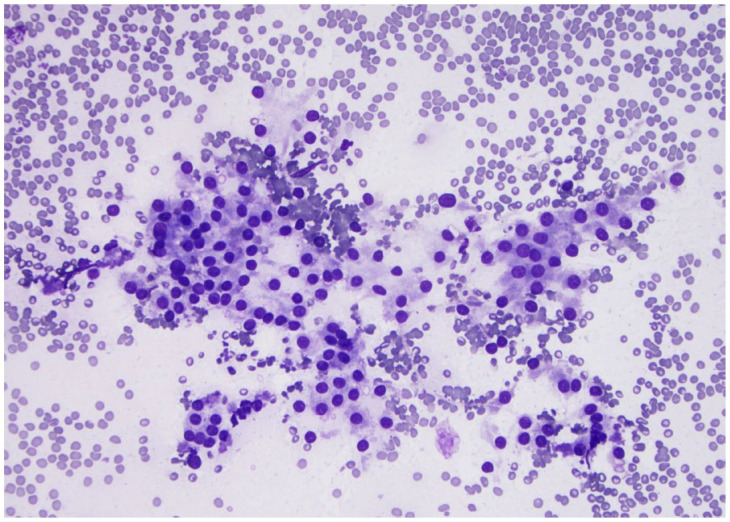
Monolayer sheet of loosely cohesive benign follicular cells (×200, Diff-Quik stain).

**Figure 13:**
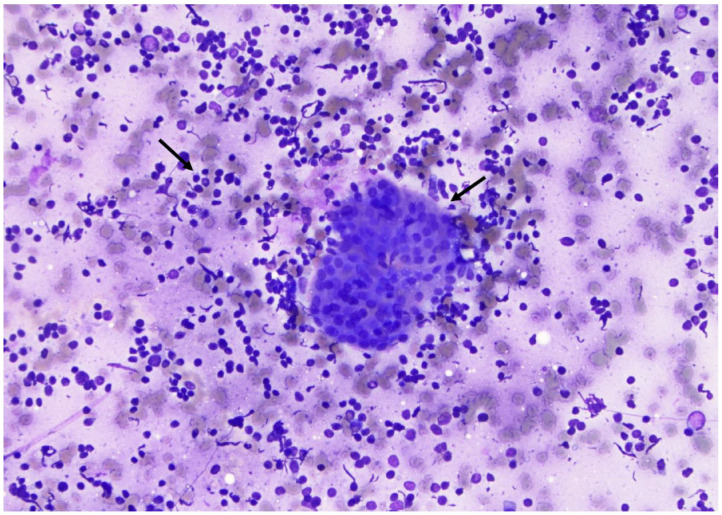
One monolayer sheet of cells with slight overlapping and numerous lymphocytes in the background (arrows) suggestive of benign lymphocytic thyroiditis (×200, Diff-Quik stain).

**Figure 14:**
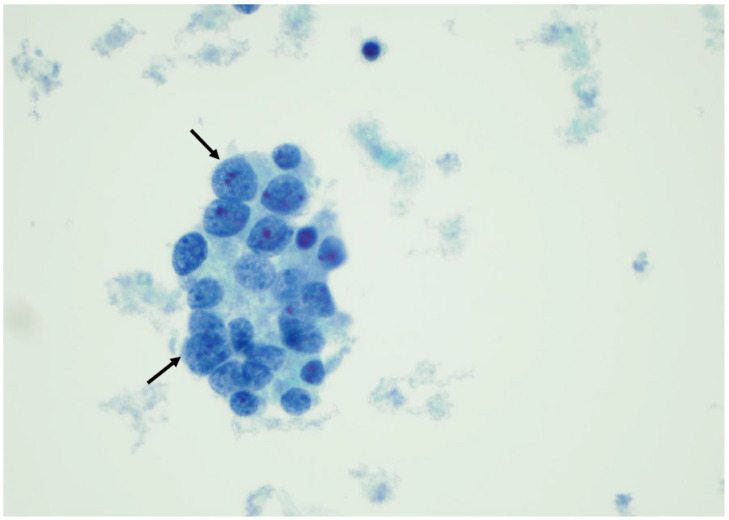
Atypia of undetermined significance—nuclear atypia. Note the enlarged nuclei with the prominent nucleoli (arrows). These changes are not typical for a papillary carcinoma but concerning in the case of low cellularity (×200, Papanicolaou stain).

**Figure 15:**
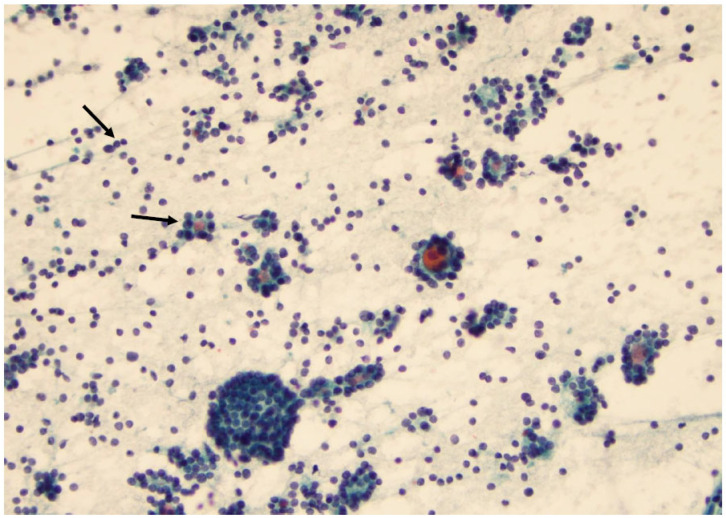
A follicular neoplasm is characterized by numerous microfollicular arrangements (arrows), as seen in this slide, with high cellularity (×200, Papanicolaou stain).

**Figure 16:**
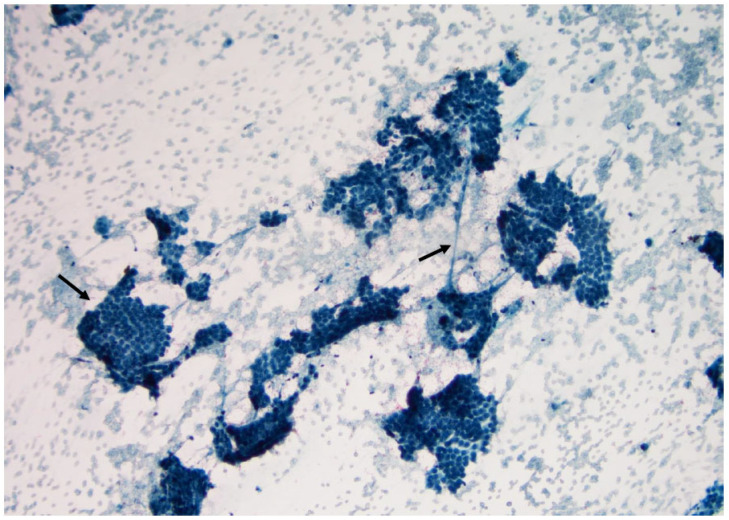
Papillary thyroid carcinoma: note the high cellularity, the large sheets of cells with distinct borders (left arrow), and the naked capillary blood vessel (right arrow) (×200, Papanicolaou stain).

**Figure 17:**
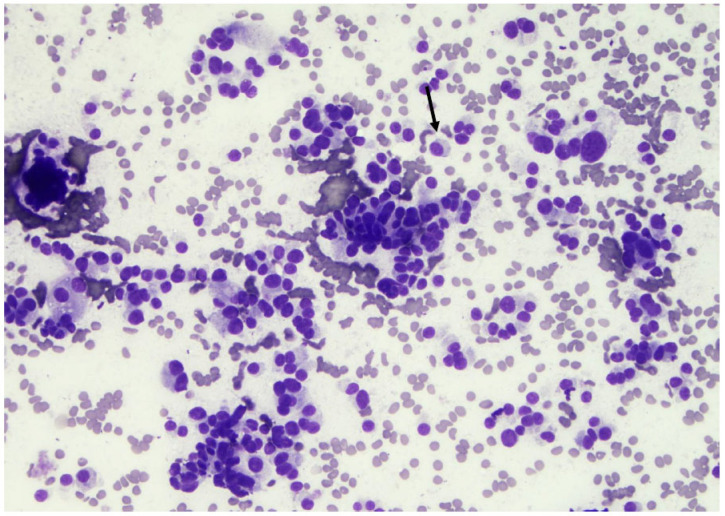
Medullary thyroid carcinoma is characterized by small- and medium-sized cells with some enlarged eccentric nuclei. Some of these cells have two or three nuclei (arrow) (×200, Diff-Quik stain).

**Figure 18:**
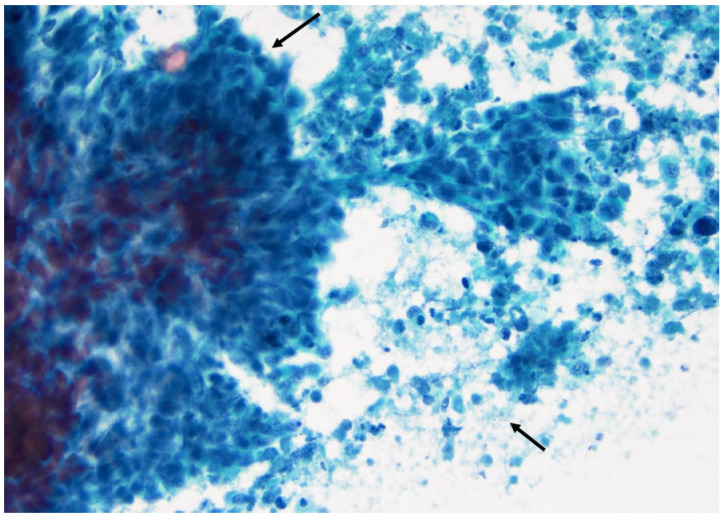
Anaplastic carcinoma is characterized by large three-dimensional sheets of large and pleomorphic cells. Note some necrosis is visible (arrow below) (×200, Papanicolaou stain).

In the updated *BSRTC*, the practice of offering two choices for category names, as seen in the 2017 version, has been eliminated. The current recommendation is that a single term be used for each category, e.g., “Nondiagnostic” in place of “Nondiagnostic/Unsatisfactory”; “Atypia of Undetermined Significance (AUS)” instead of “Atypia of Undetermined Significance/Follicular Lesion of Undetermined Significance (AUS/FLUS)”; and “Follicular Neoplasm (FN)” in lieu of “Follicular Neoplasm/Suspicious For a Follicular Neoplasm (FN/SFN)”. In the last category, the term “suspicious for” has been removed to eliminate confusion with the category “Suspicious for malignancy”. Once again, it is emphasized that the pathologist’s reports should not be assigned numerical values (e.g., Category 2 or Category 5). Across all categories, it is imperative a more precise diagnosis be provided. For example, within the “Benign” category, specifying whether it is a follicular nodule or thyroiditis is necessary. Similarly, within the “Malignant” category (Category 6), specifying the specific type of cancer is essential (Figure 12, Figure 13, Figure 14, Figure 15, Figure 16, Figure 17 and Figure 18). Additionally, the “Follicular Neoplasm” category now encompasses the oncocytic variant, which should also be clearly specified (Table 2).

The category AUS is now divided into two sub-categories: AUS—nuclear atypia and AUS—other. This division into two sub-categories is based on the differences in their ROM, with AUS—nuclear atypia displaying a twofold higher ROM compared to AUS—other.

**The AUS—nuclear atypia** sub-category encompasses cases characterized by nuclear atypia suggestive of papillary thyroid carcinoma (PTC) but lacking sufficient evidence to warrant a “Suspicious for Malignancy” diagnosis. Within the AUS—nuclear atypia sub-category, the following former sub-categories of *The BSRTC* 2017 have now been consolidated: 

Focal nuclear atypia;

Extensive but mild nuclear atypia;

Atypical cyst-lining cells;

“Histiocytoid” cells;

Nuclear and architectural atypia (may be suggestive of NIFTP).

**The AUS—other** sub-category pertains to cases displaying architectural alterations as well as oncocytic nodules within the context of a Thyroid follicular nodular disease (TFND) or Hashimoto’s thyroiditis. Within the AUS—other sub-category, the following former sub-categories of *The BSRTC* 2017 are now consolidated: 

Architectural atypia (focal microfollicles or a majority of microfollicles in a sub-optimal FNA);

Oncocyticoncocyte atypia (oncocytes with abundant colloid; in the context of Hashimoto thyroiditis; in a multinodular thyroid gland; in a sparsely cellular FNA);

Atypia, when not otherwise specified (NOS);

Atypical lymphoid cells, rule out lymphoma

The ROM for each sub-category is presented above (Table 1).

Information regarding the risk of malignancy (ROM) for each category has undergone extensive refinement, taking into consideration recent studies and addressing potential biases, which are particularly notable in certain categories, such as AUS. This bias arises because a significant portion of surgical interventions occur in AUS nodules when accompanied by additional data that arouse suspicion of malignancy. Conversely, AUS nodules lacking clinical or radiological data suggestive of malignancy are typically not subjected to surgical treatment and subsequent monitoring. As a result, surgical confirmation is no longer the sole reference point, and diverse ROM figures are now proposed, incorporating a comprehensive spectrum of available data. This encompasses follow-up information, ancillary techniques, and the recognition of the NIFTP entity, which is a notably indolent tumor frequently diagnosed as “AUS–nuclear atypia”.

The risk of malignancy (ROM) for the “Nondiagnostic” and “AUS” categories is relatively high at 13% and 22%, respectively, in contrast to the estimated ROM from 2017 for these categories [11]. This elevated ROM is a direct outcome of the surgical bias mentioned earlier. For AUS, the ROM is notably higher within the AUS—nuclear atypia subset than the AUS—other subset, ranging from 36% to 44% for AUS with nuclear atypia and 15% to 23% for AUS with other patterns [12]. However, these figures still require precise assessment. Both standard and diverse ROM values are proposed, drawing upon all presently available data. The intention behind suggesting these ROM values is to equip clinicians with necessary information to prevent unwarranted lobectomy or, conversely, to guide surgical intervention. The latest *BSRTC* has also incorporated the terminology from the 2022 WHO Classification of Thyroid Neoplasms and endeavored to employ the same language. As such, the term “differentiated high-grade thyroid carcinoma (DHGTC)” is introduced and discussed. Within the context of a multinodular thyroid gland, the suggested term is “consistent with thyroid follicular nodular disease.” This presents a challenge for radiologists tasked with identifying the nodule(s) warranting fine-needle aspiration (FNA), typically involving the largest nodule(s) and those exhibiting suspicious ultrasonographic attributes.

**The management of the patient**

Additional findings encompassing clinical data, US imaging, and other imaging studies if necessary, alongside ancillary tests, have the potential to influence the approach to assessing a thyroid nodule. In terms of nodule management, especially when various options are available, the decision-making process is anchored in the Bethesda category while also being guided by clinical information and ultrasound characteristics. At times, patient preferences come into play, leading to the development of intricate algorithms, as exemplified in recent propositions by the SFE (Société Française d’Endocrinologie-Consensus 2022) [13]. Additionally, the integration of ancillary techniques, including immunocytochemistry (ICC) and molecular testing, further enriches these algorithms.

**Immunocytochemistry (ICC)**

For certain diagnoses, ICC is highly valued in the context of thyroid nodules. It proves useful in identifying conditions such as medullary thyroid carcinoma (MTC) through calcitonin ICC, as well as for detecting metastases, lymphoma, and rare primitive tumors like paraganglioma. As a result, the new *BSRTC* addresses ICC. Nonetheless, ICC is not recommended for refining the classification of certain follicular-cell-derived tumors, with molecular testing being considered more discerning. Nevertheless, in regions like Europe where these tests might not be readily available or cost-effective, several studies have indicated that using panels of two or three antibodies could significantly enhance diagnostic accuracy, especially with a notably high Negative Predictive Value (NPV) ranging from 97% to 99%. Among these panels, the combination of CK19/HBME1/Gal 3 [14], CK19/HBME1/TPO, and Gal 3/HBME1/TPO demonstrated the highest Positive Predictive Value (PPV) when paired with an exceptional NPV. Notably, the most efficient two-antibody panel, maintaining the same NPV as the three-antibody panel, seems to be HBME1/TPO [15]. Moreover, the availability of the BRAF V600E antibody (clone VE1) has led to the publication of numerous studies reporting its utilization. However, only a positive BRAF V600E outcome is considered valid, as the majority of follicular variant of papillary thyroid carcinoma (FVPTC) cases are BRAF V600E-negative. 

**Molecular testing**

Several validated molecular diagnostic tests are now accessible and prove particularly valuable, especially within the AUS/FN/SM categories. These tests offer an enhanced approach to risk stratification for thyroid nodules. However, it is important to note that the efficacy of this risk stratification varies across different tests, as highlighted in the recent 2022 SFE consensus [16]. This consensus compared the performance metrics (NPV/PPV/sensitivity/specificity) of the primary four tests available: the seven-gene panel, ThyroSeq^®^ Genomic Classifier, Afirma^®^ Genomic Sequencing Classifier and Xpression Atlas, and ThyGeNEXT and ThyraMIR^®^.

Furthermore, the risk stratification process must be evaluated in conjunction with the cytological category, underscoring the pivotal role of morphological diagnosis. Particularly within follicular-cell-derived tumors, aside from the highly specific BRAF V600E and RET/PTC mutations, the significance of other markers is relatively limited. For instance, as exemplified in a recent study by V Marotta et al. [17], RAS mutations, spanning a broad spectrum from benign to malignant tumors, contribute significantly to the improved classification and understanding of these tumors. However, these mutations alone cannot serve as definitive “rule in or rule out” testing outcomes; they necessitate analysis alongside a panel of other genetic anomalies. Consequently, molecular tests do not provide a straightforward “black and white” outcome but need to be incorporated into intricate algorithms that encompass the Bethesda categories, clinical data, and the specific performance characteristics of the applied molecular test, given that their results are not uniformly equivalent, as elaborated on above (Table 3).

## 4. Conclusions

The new WHO 2022 classification, encompassing recent entities with a notably low risk of malignancy such as NIFTP, as well as more aggressive cancers like differentiated high-grade follicular carcinoma, is poised to significantly assist clinicians in tailoring appropriate therapeutic approaches. The updated 2023 *BSRTC*, which takes into consideration a combination of clinical, radiological, histological, and molecular data for precise risk of malignancy (ROM) assessment, is expected to guide the customization of patient management. Indeed, achieving the utmost precision in diagnosis is imperative and involves the convergence of cytological findings, ultrasonography, and ancillary techniques, before arriving at decisions regarding follow-up or surgery for thyroid nodules. These decisions align with meticulously detailed algorithms outlined by both American and European guidelines.

## Figures and Tables

**Table 1:** Usual management of patients based exclusively on cytology.

Diagnostic Category	ROM Ave% (Range)	Usual Management
**Nondiagnostic**	**13** (5–20)	Repeat FNA with ultrasound guidance
**Benign**	**4** (2–7)	Clinical and sonographic follow-up
**Atypia of Undetermined** **Significance**	**22** (13–30)	Repeat FNA, molecular testing, diagnostic lobectomy, or surveillance
**Follicular Neoplasm**	**30** (23–34)	Molecular testing, diagnostic lobectomy
**Suspicious for Malignancy**	**74** (67–83)	Molecular testing, lobectomy or near-total thyroidectomy
**Malignant**	**97** (97–100)	Lobectomy or near-total thyroidectomy

**Table 2:** Categories and diagnoses.

I. Nondiagnostic
Cyst fluid only
Virtually acellular specimen
Other (obscuring blood, clotting artifact, drying artifact, etc.)
**II. Benign**
Consistent with follicular nodular disease (includes adenomatoid nodule, colloid nodule, etc.)
Consistent with chronic lymphocytic (Hashimoto) thyroiditis in the proper clinical context
Consistent with granulomatous (subacute) thyroiditis
Other
**III. Atypia of Undetermined Significance**
Specify type: AUS—nuclear atypia or AUS—other
**IV. Follicular Neoplasm**
**V. Suspicious for malignancy**
Suspicious for papillary thyroid carcinoma
Suspicious for medullary thyroid carcinoma
Suspicious for metastatic carcinoma
Suspicious for lymphoma
Other
**VI. Malignant**
Papillary thyroid carcinoma
High-grade follicular-derived carcinoma
Medullary thyroid carcinoma
Undifferentiated (anaplastic) carcinoma
Carcinoma with mixed features (specify)
Metastatic malignancy
Non-Hodgkin lymphoma
Other

**Table 3:** Main-but not exhaustive- molecular changes.

Histology	Follicular Adenoma	HTT	WDT-UMP	FV-UMP	NIFT-P	PTC Classic	IEFVPTC	Tall cell PTC	Hobnail PTC	Solid PTC	Diffuse Sclerosing PTC	FTC	Oncocytic Carcinoma	PDTC/ DHGTC	Anaplastic Carcinoma
RAS mutations	Found *	Not found	Found *	Found *	Found **	Not found	Found **					**Found**		**Found**	
PAX8:PPARG	Found *		Rare	Found *	Found **		Found *								
Thada fusions	Found *				Found **		Rare								
PAX8: GLIS1/GLIS3 fusions		Found **													
NTRK fusions										**Found**					
BRAF V600E		Not found	Not found	Not found	Not found	Found **	Rare	Found **	Found **	Found *	Found *			**Found**	**Found**
TERT promoter								**Found**	**Found**					**Found**	**Found**
TP53								**Found**	**Found**					**Found**	**Found**
RET rearrangements						Found **	Rare			**Found**	Found **				
Mitochondrial DNA mutations													**Found**		
Chromosomes losses													**Found**		

* in a low percentage 10–20%; ** in a high percentage 40% to up 70%.

## Data Availability

Data will be made available upon request.
